# Involvement of Platelet–Tumor Cell Interaction in Immune Evasion. Potential Role of Podocalyxin-Like Protein 1

**DOI:** 10.3389/fonc.2014.00245

**Published:** 2014-09-11

**Authors:** Laura Amo, Estíbaliz Tamayo-Orbegozo, Natalia Maruri, Cristina Eguizabal, Olatz Zenarruzabeitia, Marta Riñón, Arantza Arrieta, Silvia Santos, Jorge Monge, Miguel Angel Vesga, Francisco Borrego, Susana Larrucea

**Affiliations:** ^1^Regulation of the Immune System Group, BioCruces Health Research Institute, Hospital Universitario Cruces, Barakaldo, Spain; ^2^Basque Center for Transfusion and Human Tissues, Galdakao, Spain; ^3^Immunopathology Group, BioCruces Health Research Institute, Hospital Universitario Cruces, Barakaldo, Spain; ^4^Ikerbasque, Basque Foundation for Science, Bilbao, Spain

**Keywords:** platelet–tumor interaction, selectin ligands, podocalyxin, immune evasion

## Abstract

Besides their essential role in hemostasis and thrombosis, platelets are involved in the onset of cancer metastasis by interacting with tumor cells. Platelets release secretory factors that promote tumor growth, angiogenesis, and metastasis. Furthermore, the formation of platelet–tumor cell aggregates in the bloodstream provides cancer cells with an immune escape mechanism by protecting circulating malignant cells from immune-mediated lysis by natural killer (NK) cells. Platelet–tumor cell interaction is accomplished by specific adhesion molecules, including integrins, selectins, and their ligands. Podocalyxin-like protein 1 (PCLP1) is a selectin-ligand protein in which overexpression has been associated with several aggressive cancers. PCLP1 expression enhances cell adherence to platelets in an integrin-dependent process and through the interaction with P-selectin expressed on activated platelets. However, the involvement of PCLP1-induced tumor–platelet interaction in tumor immune evasion still remains unexplored. The identification of selectin ligands involved in the interaction of platelets with tumor cells may provide help for the development of effective therapies to restrain cancer cell dissemination. This article summarizes the current knowledge on molecules that participate in platelet–tumor cell interaction as well as discusses the potential role of PCLP1 as a molecule implicated in tumor immune evasion.

## Introduction

The metastasis to distant organ sites depends on the interaction between tumor cells and the microenvironment of the bloodstream and tissues, including platelets, immune system cells, endothelial cells, stromal cells, and extracellular matrix (1). The participation of platelets and coagulation in tumor metastasis has been observed for a long time (2). Moreover, thrombocytosis and thrombosis have been associated with poor prognosis in various tumor types (3). These observations and the finding that suppression of platelet–tumor cell aggregates with antiplatelet agents inhibits experimental metastasis point to an important role of platelets in the development of tumor metastasis (4). During platelet–tumor cell interaction, the communication between both cell types is bilateral, as tumor cells express factors that trigger platelet activation and coagulation, and activated platelets promote tumor growth, metastasis, and angiogenesis (5).

The contribution of selectins and its ligands to the communication between tumor cells and platelets has been confirmed by several preclinical models (6). Here, we describe the molecules involved in platelet–tumor interaction, specially focusing on the mechanisms by which PCLP1, a transmembrane sialomucin with selectin-ligand activity, participates in platelet–tumor aggregate formation. We also hypothesize that PCLP1 might be involved in protecting tumor cell from natural killer (NK) cell-mediated cytotoxicity.

## Platelets in Tumor Progression

Metastasis is the main complication and principal cause of death in most types of cancer. The process of metastasis consists of different phases: (1) detachment of cancer cells from the primary tumor, (2) intravasation into the vascular system and transport through the bloodstream, (3) extravasation, and (4) proliferation in a distant tissue (7, 8).

During circulation through the bloodstream and vascular adhesion at distant sites, tumor cells are prone to death induced by shear stress and attack of immune cells. However, once tumor cells intravasate into blood, tumor-derived platelet agonists, such as adenosine diphosphate (ADP) and thrombin, induce platelet activation, followed by the formation of heterotypic aggregates that protect tumor cells from immune attack and physical damage (9, 10). Tissue factor expressed by tumor cells also leads to thrombin generation through the activation of the coagulation cascade that ultimately results in fibrin formation and platelet activation (11). These processes, among others, promote the development of venous thromboembolism in solid tumor and hematological malignancies, including lymphomas and myeloma, and disseminated intravascular coagulation with severe bleeding, which is frequently associated with acute leukemia (12). The relevance of tumor-induced platelet aggregation in cancer progression is highlighted by the correlation between the ability of tumor cells to form platelet aggregates and their metastatic potential.

Platelets promote tumor cell arrest within the vasculature, endothelium cell retraction, and subsequent extravasation and tissue invasion through the release of chemokines, proteolytic enzymes such as gelatinase and heparanase, various metalloproteinases, and microparticules (10). Moreover, platelets contain proangiogenic and antiangiogenic molecules within α-granules, which are selectively released depending on the stimuli and are the most important source of vascular endothelial growth factor (VEGF). The expression of the proangiogenic molecule VEGF increases substantially in patients with cancer, promoting the formation of new vessels (13, 14). Platelets also release soluble factors that increase tumor growth at sites of extravasation. Thrombin and lysophosphatidic acid (LPA) released by platelets enhance tumor cell growth and metastasis through the activation of thrombin receptor PAR1 and LPA receptor, respectively, both expressed on tumor cells (1).

## Platelets in Tumor Immune Evasion

Platelets also contribute to metastasis by protecting disseminating tumor cells from NK cell cytolytic activity as supported by the observation that the antimetastatic effect of platelet removal is abolished by simultaneous depletion of NK cells (15–17). NK cells are potent cytolytic lymphocytes of the innate immune system that play a critical role in the elimination of tumor cells. These lymphocytes recognize target cells without prior sensitization by means of signals received from activating and inhibitory receptors, being the balance between these two opposing signals, which determines the activation state of NK cells (18). Nevertheless, tumor cells have developed different mechanisms to evade NK immunosurveillance, including the modulation of NK receptor–ligand expression patterns and the secretion of immunoregulatory molecules like IDO and PGE2 or immunosuppressive cytokines like IL-10 and tumor growth factor-β (TGF-β) (19–22).

In early phases of the metastatic process, platelets may surround tumor cells and, in conjunction with fibrinogen, form a dense coat around tumor cells, providing protection from NK cell recognition and destruction (23, 24). Besides, both TGF-β and platelet-derived growth factor (PDGF) secreted by activated platelets inhibit NK cell effector functions. Particularly, it has been reported that TGF-β down-regulates the expression of the activating receptor NKG2D on NK cells after interaction with tumor cells and, to a lesser extent, NKp30, NKp44, NKp46, and NKp80 expression (25). Furthermore, TGF-β reduces IFN-γ production by NK cells and promotes epithelial–mesenchymal-like transition in various cancer cell lines (26). PDGF diminishes the interaction of NK with target cells and NK cell cytotoxic activity (27–29). Recently, it has been reported that glucocorticoid-induced TNF-related ligand (GITRL) up-regulated upon platelet activation inhibits NK cell cytotoxicity and IFN-γ production via GITR expressed on NK cells (30).

## Molecules Involved in Platelet–Tumor Interaction

Tumor cell interaction with platelets, leukocytes, and endothelium is mediated mainly by integrins and their ligands and by the binding of P-selectin with selectin ligands expressed on tumor cells.

### Integrins

Integrins are transmembrane glycoproteins composed of an α and a β subunits forming a non-covalent heterodimer that promote cell–cell and cell–extracellular matrix adhesion, and modulates multiple cellular processes such as cell migration, proliferation, and survival. Integrins become activated through a process involving an “inside-out” signal from other receptors that results in a change in the integrin structure from a low affinity conformation to a high affinity form that enables ligand binding (31).

During the hematogenous phase of metastasis, binding of soluble platelet agonists to their receptors elicit various signaling transduction events that ultimately induce inside-out signaling processes that culminate in activation of the ligand-binding function of integrin α_IIb_β_3_ (GPIIb/IIIa) and granule secretion (10). The homotypic interaction between platelets occurs via cross-linking of integrins, primarily α_IIb_β_3_, by adhesive ligands that serve as bridging proteins present in plasma or released from activated platelets, including fibrinogen, fibronectin, von Willebrand factor (vWF), and other ligands containing the arginine–glycine–aspartate (RGD) integrin-binding domain (32). Ligand binding to integrin α_IIb_β_3_ then mediates platelet aggregation and triggers “outside-in” signaling that amplifies platelet response, resulting in the stabilization of platelet adhesion and aggregation, and clot retraction.

**Figure 1:**
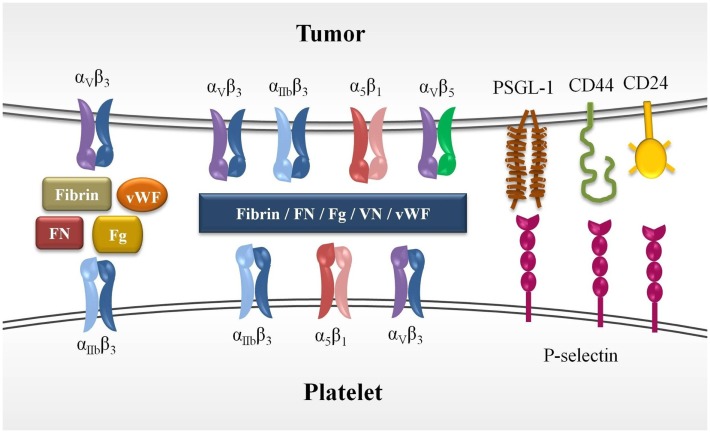
**Molecules involved in platelet–tumor cell interaction**. Platelet–tumor cell aggregates are formed (1) by cross-linking of platelet integrins, primarily α_IIb_β_3_ integrins, with α_v_β_3_ integrin expressed on tumor cells through their ligands, which act as bringing molecules (Fg, FN, fibrin, and vWF), (2) by interaction of platelet P-selectin with its selectin-ligands expressed on tumor cells (PSGL-1, CD44, CD24). The ectopic expression of megakaryocytic genes in various tumor cells leads to the expression of functional α_IIb_β_3_, and therefore, the heterotypic interaction between platelets and tumor cells may take place through cross-linking of this integrin. Other integrins has also been suggested to participate in platelet–tumor aggregates formation. FN, fibronectin; Fg, fibrinogen; vWF, von Willebrand factor; VN, vitronectin.

Platelet–tumor cell aggregates result from cross-linking of platelet integrins, primarily α_IIb_β_3_, with integrins expressed on tumor cells, such as α_v_β_3_, by the above mentioned adhesive ligands (33). The relevance of α_IIb_β_3_ and α_v_β_3_ interaction in tumor metastasis has been demonstrated in *in vivo* models, which showed a decrease of pulmonary metastasis following inhibition of α_v_β_3_ with a specific monoclonal antibody, an effect that was significantly reduced after platelet depletion (34). α_IIb_β_3_ and α_v_β_3_ integrins also support the arrest of tumor cells to the endothelium of metastatic sites. Other integrins such as α_5_β_1_ and α_3_β_1_ as well as the adhesive ligands vitronectin and laminin have been implicated in platelet–tumor interaction, tumor adhesion, and metastasis (35) (Figure 1).

### Selectins

Selectins are cell-surface adhesion molecules with a carbohydrate-binding domain that bind with low affinity to sialylated and fucosylated glycan structures present on selectin ligands and induce integrin activation. Several studies have shown that selectins may transduce outside-in signals upon interaction with their ligands (36, 37). In cancer cell interactions, selectins expressed on platelets, leukocytes, and endothelium bind to selectin ligands present on tumor cells, leading to the formation of platelet–tumor-leukocyte aggregates and tumor cell arrest in the microvasculature (38).

The selectin family consists of three molecules with selective cell distribution. P-selectin is stored in the alpha and dense granules of platelets and in the Weibel–Palade bodies of endothelial cells and translocated to surface upon cellular activation by agonists. P-selectin binds to a variety of human cancer cells, such as colon, lung, and breast cancer, as well as melanoma and neuroblastoma (39). Platelets from P-selectin-deficient mice exhibit a reduced interaction with tumor cells, resulting in a marked decrease of metastasis and reflecting the importance of this protein in tumor progression (40, 41). L-selectin, a molecule constitutively expressed on the majority of leukocytes, enables leukocyte homing to lymphoid organs and extravasation into inflamed tissues. This molecule facilitates tumor metastasis and acts synergistically with P-selectin (42). Although E-selectin, expressed on endothelial cells, has not been implicated in platelet–tumor interaction, it participates in the homing of metastatic cancer cells to distant organs (43).

### Selectin ligands

The tetrasaccharide sialyl-Lewis^x^ (sLex) and its isomer sialyl-Lewis^a^ (sLea) recognized by selectins are located in terminal chains of glycolipids and N-/O-glycoproteins displayed on selectin ligands. High cell-surface expression of sLex and sLea or altered glycosylation on tumor cells has been associated with tumor progression and metastasis (44). Selectin ligands are mainly sialylated, fucosylated, sulfated glycans localized on tumor cell mucins, that is, heavily glycosylated proteins with O-linked oligosaccharides.

Several mucin-like molecules with P-selectin ligand activity have been identified. P-selectin glycoprotein ligand-1 (PSGL-1) is a sialylated mucin-type disulfide-linked homodimer expressed on most leukocytes, which presents a high-affinity binding to P-selectin and is essential for the homing of leukocytes to tissue. PSGL-1 has also been described as a P-selectin ligand on lung cancer and myeloma cells, and an E-selectin ligand on prostate tumor cells (45–47). CD24 modified by sLex serves as the major P-selectin-reactive ligand on the surface of breast cancer cells and its enhanced expression is related to cancer progression and poor prognosis (48, 49). CD44 variant isoforms (CD44v) act as E-/L-/P-selectin ligands on colon cancer cells and as E-selectin ligand on breast cancer cells (50, 51). Podocalyxin-like protein 1 (PCLP1), a cell-surface sialomucin expressed in a wide range of normal cell as well as in various types of cancer, has been also associated with metastasis (52).

## PCLP1, a Selectin Ligand Involved in Platelet–Tumor Interaction

Podocalyxin-like protein 1 is a cell-surface glycoprotein that belongs to the CD34 family of sialomucins (53). Initially identified on kidney podocytes, PCLP1 is widely expressed on vascular endothelium, mesothelial cells, hematopoietic stem and progenitor cells, a subset of neuronal cells, and platelets (54–58). PCLP1 is attached to the actin cytoskeleton through its association with ezrin, a protein that regulates cell adhesion, motility, and survival (59). In renal glomerular podocytes, PCLP1 functions as an anti-adhesive molecule, which plays a vital role in maintaining opened the filtration slits due to its highly negative charge, as evidenced by the observation of severe renal malformation and anuria in PCLP1-deficient mice (60). On the contrary, in high endothelial venules, PCLP1 serves as a pro-adhesive molecule participating in the tethering and rolling of lymphocytes via interaction with L-selectin (53). During embryonic development, PCLP1 expression by mesothelial cells is required for the retraction of the gut from the umbilical cord (60). In the hematopoietic system, PCLP1 participates in cell migration to distant hematopoietic tissues (56). PCLP1 is also involved in neuronal development and synapse formation (61).

The overexpression of PCLP1 has been associated with a more aggressive phenotype and poor prognosis in numerous types of cancer, including breast, colorectal, prostate, bladder, ovarian cancer, renal carcinoma, and oral squamous cell carcinoma, which suggests an important role of this protein in cancer progression and metastasis, although the underlying mechanisms are poorly understood (62–69). PCLP1 is also overexpressed in blasts of acute leukemia, testicular tumor, hepatic carcinoma, astrocytic tumor, and small cell lung carcinoma (70–74). PCLP1 increases *in vitro* migration and invasion of tumor cells through its interaction with ezrin in breast cancer cells (75) and promotes invadopodia formation and metastasis by activating Rac1/Cdc42/cortactin signaling cascade (76). Recently, it has been demonstrated that PCLP1 expression in A549 human lung carcinoma cell line is increased during TGF-β-induced epithelial–mesenchymal transition and regulates the molecular changes associated with this process (77).

Although data support a correlation between PCLP1 expression and enhanced binding of metastatic tumor cells to selectins, evidences showing a functional role of PCLP1 as a selectin-ligand in cancer are limited. Recent reports show that PCLP1 expressed on colon carcinoma or pancreatic cells display E- and L-, but not P-, selectin-mediated adhesion under flow conditions (78, 79). The first study was conducted by perfusing E- and P-selectin-expressing Chinese Ovarian (CHO) cells and L-selectin-expressing human peripheral blood lymphocytes over purified PCLP1 protein derived from colon carcinoma cells and by cell-free flow-based adhesion assays using PCLP1-coated microspheres (78). Interestingly, the results proved that carbohydrate determinants involved in binding of PCLP1 expressed in colon carcinoma cells to L- and E-selectins differ from that displayed by PCLP1 expressed in high endothelial venules. The second report used PCLP1-knockdown pancreatic cells perfused over immobilized E- and L-selectin, demonstrating that PCLP1 is functionally relevant in tumor cell-selectin interaction (79).

The role for PCLP1 in promoting cell–platelet interactions is supported by a study performed in Tera-1 cell line, derived from an embryonal carcinoma of the testis expressing high levels of PCLP1, and in CHO cells ectopically expressing PCLP1 (80). This report provided evidence that PCLP1 increases the adhesion of cells to fibrinogen-immobilized platelets in a sialylation-dependent manner, further enhanced by activation of platelets by agonist. PCLP1-induced adhesion is partially inhibited by blockers of P-selectin and by an inhibitor of integrins α_v_β_3_/α_v_β_5_, and almost totally restrained by RGD peptide, a potent inhibitor of many integrin–ligand interactions, indicating the contribution of these adhesion molecules to this process. Besides, PCLP1 colocalizes with P-selectin expressed on activated platelets at cell contact sites. Another report demonstrated that PCLP1 increases cell adherence to human vascular endothelial cells, an effect that is amplified after stimulation of these cells with thrombin or histamine (81). Furthermore, PCLP1 enhances cell adhesion to immobilized fibronectin as well as cell spreading and migration. PCLP1-mediated cell adhesion to fibronectin is also dependent on the activity of integrins, as it is prevented by RGD peptides or by inhibitors of α_v_β_3_ integrin, whereas cell spreading is controlled by α_5_β_1_ integrin.

**Figure 2:**
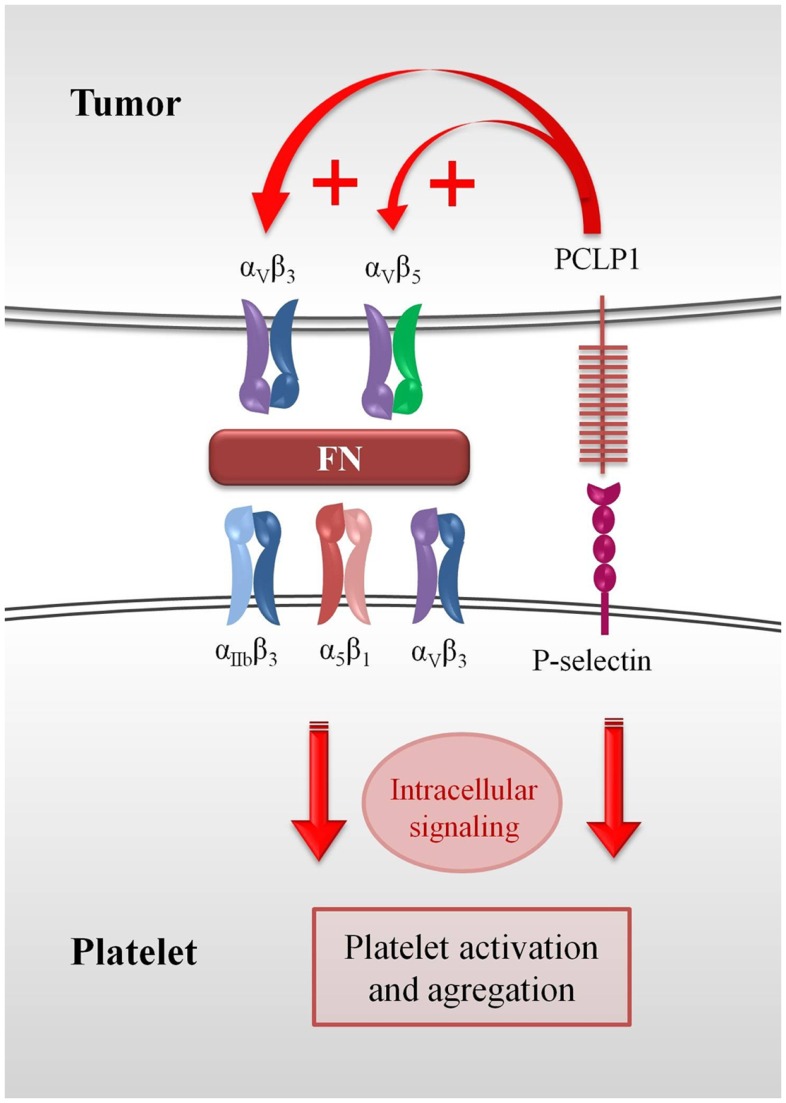
**Role of PCLP1 in platelet–tumor cell interaction**. The ability of PCLP1 to potentiate cell binding to fibronectin through the activation of integrins could increase the interaction of platelets with tumor cells. Fibronectin would act as a bridge molecule between α_v_β_3_ or α_v_β_5_ expressed on tumor cells and α_IIb_β_3_, α_v_β_3_, and α_5_β_1_ integrins expressed on platelets. This interaction might trigger outside-in signals that would potentiate the activation state of platelets, eventually leading to the stabilization of the hetero-aggregates. Moreover, PCLP1 binding to P-selectin could also result in the generation of intracellular signaling that would reinforce the hetero-aggregates.

Fibronectin, a protein that forms part of the extracellular matrix, is also abundant in plasma and mediates platelet aggregation by binding β_3_ integrins and tethering adjacent platelets (82, 83). It has been reported that incorporation of plasma fibronectin into fibrin clots enhances metastasis to the lungs (84). Thus, the ability of PCLP1 to potentiate cell binding to fibronectin through the activation of integrins could increase the interaction of platelets with tumor cells, fibronectin acting as a bridge molecule between α_v_β_3_ or α_v_β_5_ expressed on tumor cells and α_IIb_β_3_, α_v_β_3_, and α_5_β_1_ integrins expressed on platelets. Subsequently, the interaction between fibronectin and integrins might trigger outside-in signals that would potentiate the activation state of platelets, thereby stabilizing the aggregates. Other adhesive molecules such as fibrinogen, vitronectin, fibrin, and vWF could also serve as bridge proteins in this process (Figure 2).

Although the signaling triggered in platelet by the interaction of P-selectin with PCLP1 has not been explored so far, it is known that selectins may transduce outside-in signals delivered by selectin ligands through their cytoplasmic tail (36). In that way, binding of PSGL-1 to P-selectin expressed on platelets induces calcium influx and a signaling cascade that triggers α_IIb_β_3_ activation, microaggregates generation, and thrombus formation (85, 86). Platelet activation is followed by the phosphorylation of tyrosine, serine, threonine, and histidine residues on the cytoplasmic tail of P-selectin (87–89). Similarly, the interaction of PCLP1 with P-selectin might induce outside-in signals that could ultimately lead to enhanced expression of adhesion molecules on platelets that would reinforce platelet–tumor cell–leukocytes aggregates, creating a positive feedback loop.

As mentioned previously, PCLP1 is also expressed on activated platelets. Mice overexpressing human PCLP1 on platelets exhibit a phenotype characterized by decreased bleeding time and enhanced platelet aggregation after agonist stimulation (90). Inversely, the deletion of *pclp1* gene in mouse platelets produce a prolonged bleeding time, decreased platelet aggregation, decreased *in vivo* thrombosis, and reduced adherence of platelets to fibrinogen under flow, all this pointing to a role of PCLP1 in the control of hemostasis (91). Thereby, PCLP1 expressed on platelet could strengthen the heterotypic conjugates formation during hematogenous metastasis and promote their retention to the vessel wall of target organs by enhancing cell adhesion to fibronectin localized on subendothelial matrix, which is exposed following endothelial cell retraction at metastatic sites.

Considering the aforementioned observations, we hypothesize that PCLP1 could have a role in the escape of tumor cells from the immune surveillance by inducing platelet–tumor interaction. PCLP1 might participate in tumor cell evasion by fostering the formation of a physical barrier to NK cell-mediated cytotoxicity comprised of platelets and adhesive proteins through the mechanisms described above. Moreover, PCLP1 contribution to platelet–tumor interaction with vascular endothelial cells may result in a decreased permanence of hetero-aggregates in the bloodstream, thereby diminishing their exposure to NK cells.

## Future Perspectives

The evidence provided by the literature suggests that an anti-PCLP1 antibody aimed at disrupting platelet–tumor aggregation could be useful as a therapeutic drug for the treatment of PCLP1-expressing cancers. As PCLP1 expression has also been reported in normal tissues, including neuron, podocytes, and hematopoietic stem cells, antibodies that display cross-reactivity with normal cells may not be appropriate as therapeutic agents. Given that selectin-binding determinants exhibited on PCLP1 expressed in tumor cells are different from those described in normal cells (78), an antibody recognizing specific glycan epitopes displayed on tumor cells might avoid undesirable side effects. In addition to antibodies, alternative antagonists may be used such as peptides that block PCLP1–selectin interaction, specific aptamers, which are oligonucleotides displaying high affinity and specificity for target molecules, and glycomimetic compounds, synthetic oligosaccharide analogs that mimic native carbohydrates.

A systematic study of PCLP1 contribution to the interaction of platelet with different tumor types has not been addressed to date. The functional properties of PCLP1 as an adhesive protein that promotes platelet–tumor aggregates presented herein may encourage researchers to reveal yet hidden aspects of this molecule.

## Conflict of Interest Statement

The authors declare that the research was conducted in the absence of any commercial or financial relationships that could be construed as a potential conflict of interest.
